# A survey of pediatric oncology nurses’ oral health knowledge, attitudes, practices, and perceived barriers in a Singapore Tertiary Children’s Hospital

**DOI:** 10.1038/s41405-023-00130-2

**Published:** 2023-02-07

**Authors:** Ruixiang Yee, Pui Ling Chay, Melissa Mei-Yi Khor, Yvonne Siew Ling Lim, Nicole Kim Luan Lee, Wee Fang Kam, Seyad Ehsan Saffari, Mei Yoke Chan

**Affiliations:** 1 Dental Service, KK Women’s and Children’s Hospital, Singapore, Singapore; 2 Hematology/Oncology Service & Pediatric Palliative Service, KK Women’s and Children’s Hospital, Singapore, Singapore; 3 Division of Surgery, KK Women’s and Children’s Hospital, Singapore, Singapore; 4 Surgery ACP, SingHealth Duke-NUS Medical School, Singapore, Singapore; 5 Health Services & Systems Research, Duke-NUS Medical School, National University of Singapore, Singapore, Singapore

**Keywords:** Oncology, Paediatric dentistry, Special care dentistry, Dental education

## Abstract

**Aim:**

To explore oral health-related knowledge, abilities, attitudes, practices, and barriers of pediatric oncology nurses at an Asian children’s hospital.

**Methods:**

A cross-sectional study was conducted via a self-administered anonymized questionnaire. Data was analyzed to summarize knowledge, confidence, and practice behaviors.

**Results:**

All sixty-three pediatric oncology nurses responded. Fifteen participants had >80% of the knowledge questions correct. Majority (97.3%) agreed on their roles in helping patients maintain their oral health. However, 75.8% of participants felt need for training in giving oral health advice. Notably, 74.6% checked patients’ mouths at least once daily but only 57.1% felt adequately trained. Though a high proportion (>90%) of nurses felt confident to assist with oral care, only 65% would assist patients to do so; “Uncooperative patient” was the major barrier reported.

**Discussion:**

Nurses have high general awareness of importance of oral health, but had incomplete knowledge. Compared to previous studies, most (90.5%) did not find performing oral care unpleasant but other barriers might have hindered actual oral care practice.

**Conclusion:**

Nurses were motivated to assist in oral care of children with cancer but sometimes felt ill-equipped. Updated national and institution guidelines, didactic and hands-on training, and implementation of practical support could be considered.

## Introduction

Oral complications (e.g., oral ulcerations, mucositis, xerostomia and secondary infections) may happen in 90% of children with cancer as a result of their disease or treatment, impacting their quality of life and survival [1, 2]. To minimize this, it is important to optimize oral health before, during, and following cancer treatment [3], by referring newly diagnosed patients to dentists who will identify and manage any oral diseases, and advise on preventive care to practice during the course of oncology treatment [4].

However, once the oncology treatment commences, dentists often have limited or no interactions with the oncology patient and are not rightly sited to reinforce preventive oral care. The pediatric oncology patient would spend a substantial amount of time as a hospital inpatient during treatment, where oncology nurses involved in the care of the child can play a crucial role in oral assessment, oral care, and education. Furthermore, all pediatric patients are dependent on adult caregivers to some degree and it is noteworthy that about half of Singapore’s childhood cancers occurs in children below 5 years old [5]. Hence, nurses also have to step in to reinforce and guide oral care in the wards, if parents require assistance in caring for the sick child. However, studies in Ireland, Sweden, and USA showed that both adult and pediatric oncology nurses have insufficient knowledge and education in oral care, and felt uncomfortable performing oral care for patients [6–8].

Approximately 70% of Singapore’s pediatric oncology patients are diagnosed and managed at KK Women’s and Children’s Hospital (KKH). There is currently no published data on the oral health-related knowledge, attitudes, practices, and barriers of pediatric oncology nurses in Singapore. This study aimed to examine the knowledge, perceived abilities, attitudes, practices and barriers of KKH pediatric oncology nurses in meeting the oral healthcare needs of children with cancer, so to identify gaps to be addressed.

## Methods

This is a cross-sectional study conducted via a self-administered anonymized questionnaire, from 13th–27th Aug 2018. Ethical approval was obtained from the SingHealth Centralized Institutional Review Board (Reference number: 2018/2591).

Currently, there is no validated comprehensive questionnaire available in the literature for this purpose. Hence, the questionnaire (Details: Supplemental File S1) was designed by the multidisciplinary study team based on a literature search of studies involving nurses and oral healthcare [8–10], and oral healthcare guidelines for children with cancer [11, 12]. It was pre-tested on two oncology nurses from the study team and five non-oncology ward nurses, and revised so that the questions were fit for purpose and easily understood.

Five key areas covered were: (1) demographics and training history, (2) oral health knowledge, (3) attitudes and beliefs, (4) perceived practices and abilities, and (5) barriers related to oral care in pediatric oncology nursing (Table 1). Knowledge questions were in the format of true/false or multiple-choice questions. For other areas, questions were in yes/no format, or Likert scales (e.g. strongly agree, agree, neutral, disagree, strongly disagree).

**Table 1:** Key aspects explored by survey questions.

**Section A: Demographics and Training History**
a. Demographics–gender, job position, nursing experience
b. Qualifications–basic and advanced nursing training
c. Oral health training history
**Section B: Oral Health Knowledge**
a. Oral health and oncology
b. Oral habits–fluoride and toothbrushing
c. Diet habits
**Section C: Attitudes and Beliefs**
a. Self-perceived importance in identifying and referring oral conditions
b. Perceived importance of oral health in oncology patients
c. Perceived need for further training in oral care for patients
**Section D: Perceived Practices and Abilities**
a. Referring and oral assessment practices
b. Self-confidence to advise and assist with oral healthcare with their patients
c. Self confidence in diagnosis of existing oral problems
**Section E: Barriers**
a. Perceived barriers to referring patients for dental treatment
b. Perceived barriers to providing oral care for patients

All 63 nurses involved in clinical care in pediatric oncology wards were invited to participate. Invitations and participant information sheets were distributed during a team meeting. The survey was voluntary and anonymized. No respondents’ identifiers were recorded. The completion/return of the questionnaire implied consent to participate. Completed questionnaires were collected via a drop-box in the wards. The nurses were given reminders during subsequent team meetings to maximize participation. Questionnaires with at least 90% answers were considered acceptable to be included in the analysis. Continuous variables were described as mean and standard deviation, and Student’s t-test was used to compare means of independent samples; categorical variables reported as frequency and percentage. Data was analyzed using SPSS Statistics software for Windows, Version 21.0 (IBM Corp, Armonk, US). A p-value of <0.05 was considered statistically significant. All data was stored on secure systems and there was no dispersal of anonymized or averaged data beyond the study team. Data will be retained for a minimum of 7 years after the date of publication to facilitate inspection by authorized authorities.

## Results

All 63 surveys were completed and returned. All had over 90% answers completed and were deemed satisfactory to include for analysis. Most questions had 100% responders, several had 1-3 missing data; only the question “How confident are you in your ability to examine if patient experiences dysphagia?” had 19 nil responses.

### Demographics

All participants were female. Most were experienced with clinical experience ≥6years (69.8%, n = 44). Over half had ≥6years of specific experience in pediatric oncology (54.0%, n = 34). The majority were Staff Nurse grade and above; over half were either Senior Staff Nurse or Assistant Nurse Clinician (58.7%, n = 37). Over three-quarters had Bachelor of Nursing degrees (76.2%, n = 48) from countries such as Singapore, Philippines and India. Many did not have oral health-related training (58.7%, n = 37), clinical competency during nursing training (76.7%, n = 46), or Continuing Professional Education (CPE) related to oral health in past 5 years (68.3%, n = 41) (Details: Supplemental Table S1).

### Knowledge

No participant answered all knowledge questions correctly. Four (6.3%) had over 90% of questions correct (13 questions); 11 (17.4%) had more than 80% of questions correct (≥12 questions). The majority (92.1%, n = 58) did not know of any local or international guidelines on oral healthcare in pediatric oncology patients.

All participants knew that children undergoing cancer therapy can potentially develop oral complications, and that good oral hygiene is important to reduce the severity of oral mucositis. All but one (98.5%) responded correctly that caregivers should assess patients’ mouth every day during active cancer therapy. Three-quarters (75.8%, n = 47) knew that oral cavity is the most common source of sepsis in immunocompromised patients with cancer. The majority knew the best timing to refer patients is before cancer treatment (81.0%, n = 51).

The majority knew that patients should brush at least twice daily with a soft toothbrush (98.4%, n = 62), regardless of their red blood cell (87.1%, n = 54), or white blood cell levels (83.9%, n = 52). However, half (52.4% n = 33) were mistaken that toothbrushing should not continue if platelet counts are low. Moreover, not all (72.6%, n = 45) knew that a fluoride toothpaste should be used. Approximately half (55.6%, n = 35) knew that oral swabs should not be used for oral hygiene as a long-term substitute for toothbrushing.

Knowledge regarding dietary practices were poor. Only 37.1% (n = 23) knew that frequency of sugary intake is a greater risk factor for dental caries than total amount of sugary intake. Although upward trends in the overall knowledge scores were observed in nurses regardless of their specialty, after attending up to 3 hours of Continuing Professional Education (CPE) related to oral care (Table 2), this did not reach statistical significance.

**Table 2:** Comparison of years of nursing experience and Continuing Professional Education (CPE) hours with overall knowledge score.

	Knowledge scores (mean ± SD)			
	Working as a nurse	p-value	Working in a pediatric ward	p-value
≤5 years				
• 0 CPE hours • ≤ 3 CPE hours	10.2 ± 1.48 9.8 ± 2.63	0.686	9.9 ± 1.60 9.9 ± 1.95	0.984
6-10 years				
• 0 CPE hours • ≤ 3 CPE hours	9.7 ± 1.49 10.7 ± 1.38	0.199	9.9 ± 1.55 10.7 ± 1.38	0.155
≥11 years				
• 0 CPE hours • ≤ 3 CPE hours	10.3 ± 1.74 11.0 ± 0.82	0.443	10.2 ± 1.92 11.0 (n = 1)	NA

SD = standard deviation. Student’s t-test was performed to compare the means of two independent groups. No nurses declared to have CPE > 3 hours.

### Attitudes and Beliefs

All participants believed that oral hygiene is important. The majority felt they play an important role in maintaining patients’ oral health (93.7%, n = 59) and ensuring patients brush teeth at least twice daily (95.1%, n = 58). In fact, 92% (n = 57) felt it was “very or somewhat important” to help brush the child’s teeth, if the parents/patients do not do so. However, three-quarters (75.8%, n = 47) felt they needed further training in oral hygiene education.

### Perceived Practices

The nurses reflected that it was mostly doctors (85.7%, n = 54) who referred patients to dentists. Moreover, only 61.9% (n = 39) were aware of an existing in-hospital dental referral form.

**Fig. 1:**
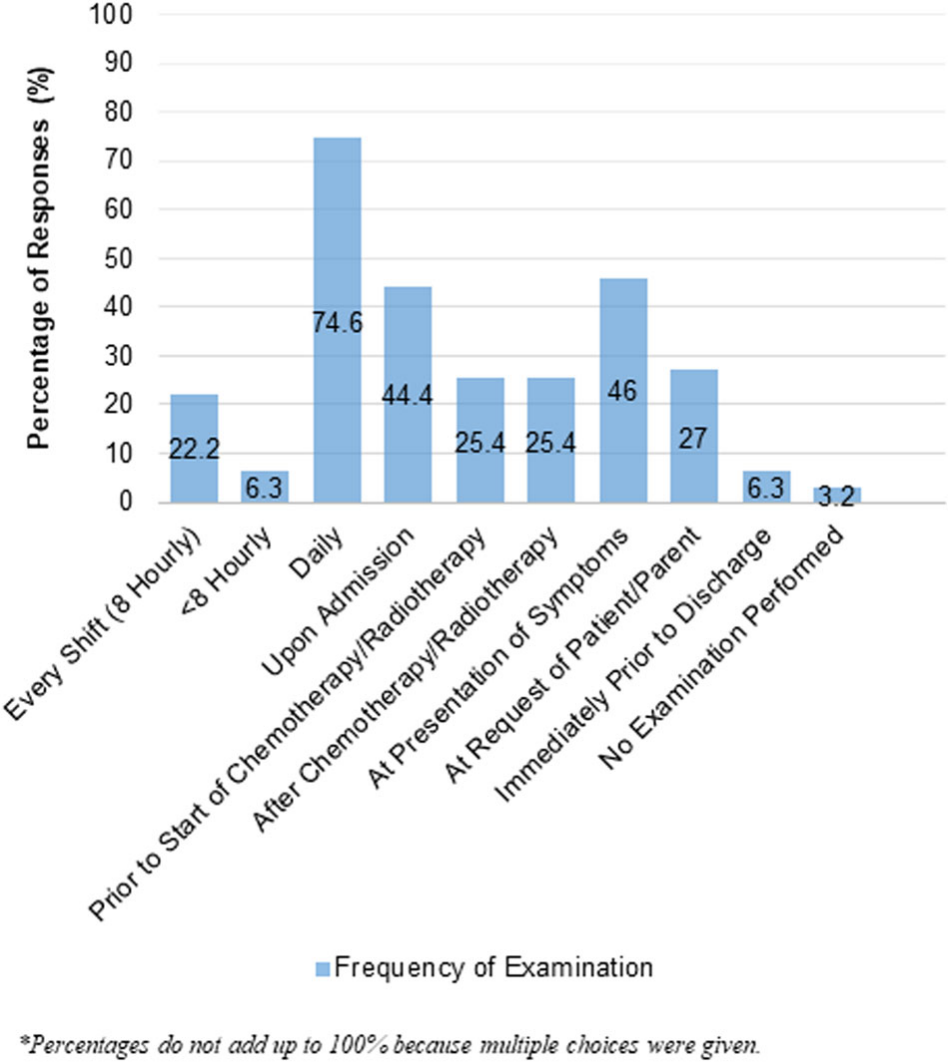
Frequency of oral examination by nurses.

The majority would check patients’ mouths at least once daily (74.6%, n = 47) (Fig. 1). Oral conditions evaluated most commonly were: oral mucositis (98.4%, n = 62), oral ulcerations (87.3%, n = 55), oral bleeding (85.7%, n = 54), swollen gums (79.4%, n = 50), and oral pain (79.4%, n = 50). Dental caries, inflamed soft tissues, dysphagia, fungal infection, clinical abscess, dental plaque, bad breath, and oral pathology were less frequently evaluated (Details: Supplemental Table S2).

Concerning toothbrushing, 85.7% (n = 54) advised patients to do so “often”/“always”; the rest “seldom”/“never did so”. Only 65% (n = 41) assisted patients to brush their teeth “often”/“always”; the rest “seldom did so”. About three-quarters would recommend fluoride toothpaste (74.2%, n = 46), 9.7% (n = 6) advised non-fluoridated toothpastes, and the rest were “not sure”/”did not” give advice (16.1% n = 10). Common oral health aids advised were foam brush, soft-bristled toothbrush, mouthwash, and lip balm (Details: Supplemental Fig. S1). About half (51.7%, n = 32) advised patients to reduce sugary intake “often”/“always”; the rest “seldom” or “never did so”.

### Perceived abilities

The majority felt comfortable (92.1% n = 58), and adequately trained (90.5%, n = 57) performing oral care, including assisting with toothbrushing, mouthwash use, and application of oral topical medications.

**Fig. 2:**
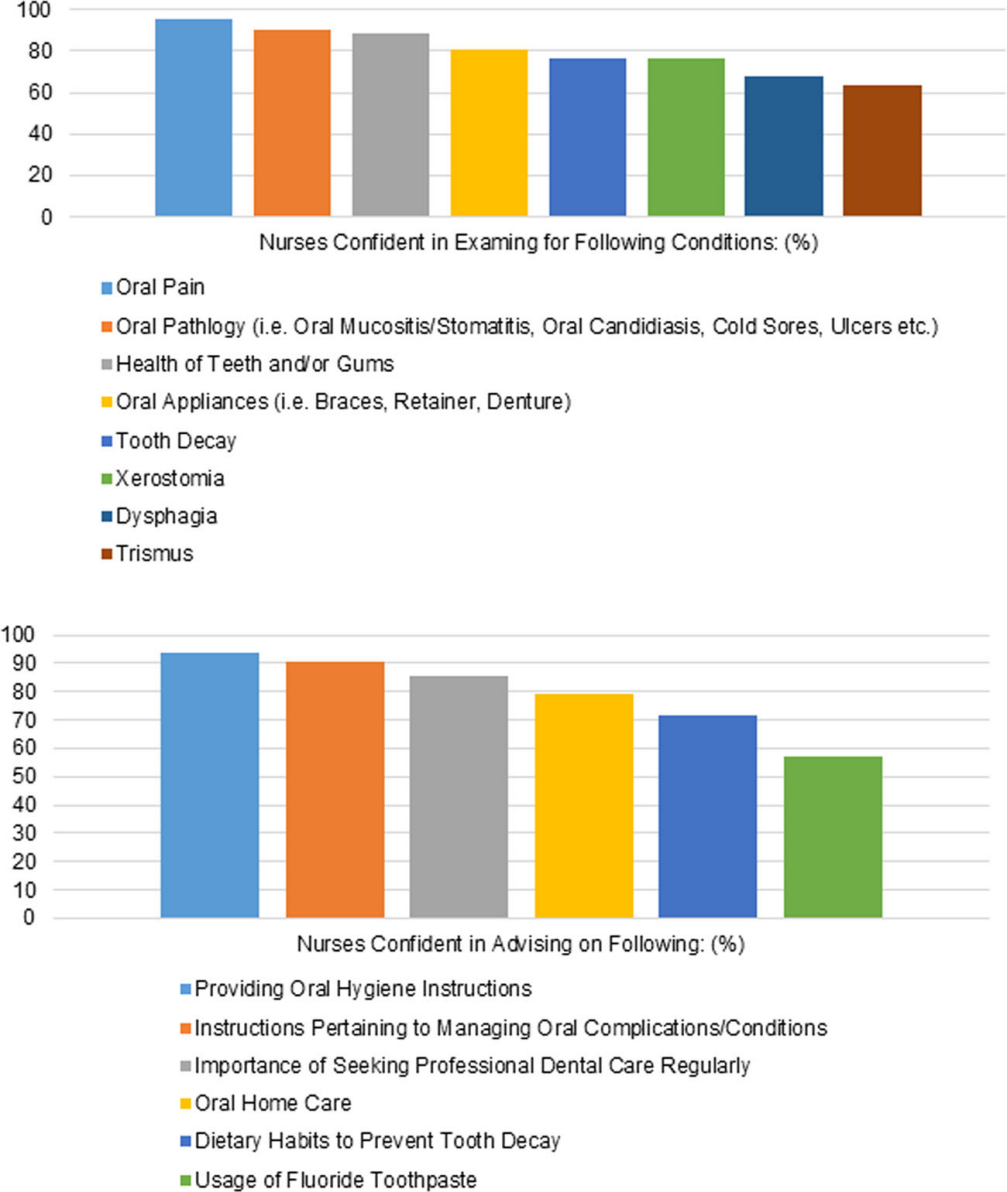
Perceived abilities of nurses in parental education and oral examination. **a** Confidence in examination of oral conditions. **b** Confidence in advising parents on oral care of child.

Over 80-90% were confident to examine for the health of teeth/gums, presence of oral pathology and oral pain, and discuss importance of regular professional dental care. Over 70-80% were confident to examine for presence of tooth decay, oral appliances or dry mouth, and providing parents with oral hygiene home care and dietary advice to prevent decay. However, only 57.1% (n = 36) were confident in advising fluoride toothpaste use. Likewise, just over 60% were confident in identifying specific problems like trismus and dysphagia (Fig. 2).

Only 65.1% (*n* = 41) felt adequately trained to give oral care instructions. Just over half (57.1%, *n* = 36) felt adequately trained to perform oral examinations.

### Barriers

**Fig. 3:**
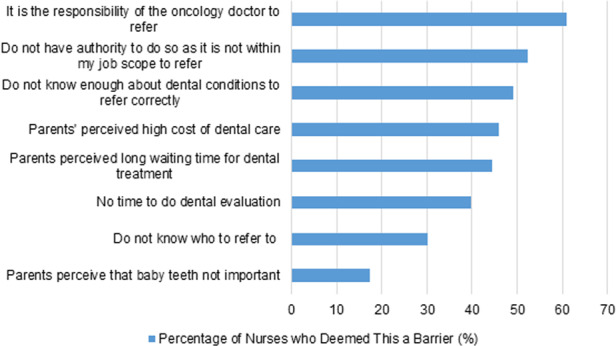
Responses to “Concerns preventing you from referring your patients to see a dentist”.

The most common barriers to dental referral reported were beliefs that it is not their responsibility or authority, followed by inadequate knowledge of dental conditions to refer for (Fig. 3).

In terms of barriers to performing oral care, the most common theme was patient-related e.g. poor patient cooperation, patient being unwell or having sore mouth. This was followed by staff and operational factors e.g. inadequate time, staff knowledge, skills, and oral care resources in ward. Most did not find oral care an unpleasant task (90.5%, n = 57) but 42.9% (n = 27) felt it was parents’ responsibility (Table 3).

**Table 3:** Responses to “Barriers that hinder me from performing oral care for the patients”, compared to a similar previous study^#^.

Barriers	Singapore Pediatric Oncology Nurses (Our study) (%)	Singapore Critical Care Nurses (Chan & Ng 2012) [9] (%)
***Staff factors***		
Lack of time	31.7	–
Inadequate staffing	25.4	11.3
Lack of oral toilet requisites	19	8.4
Lack of knowledge	17.5	3.8
Not sure what to look out for	17.5	–
I have other more important tasks	11.1	–
Doctors are the ones responsible	9.5	–
It is an unpleasant task	9.5	0.4
***Patient factors***		
Uncooperative patient	90.5	88.7
Unwell patient	41.3	28.0
Patient has a sore mouth	38.1	–
Intubated patients	11.1	11.3
***Others***	–	8.8
Parents are the ones responsible	42.9	–

^#^Multiple responses possible.

## Discussion

Limited studies on oral health-related practices and knowledge of pediatric oncology nurses exist. Surveys conducted in a conference at Pennsylvania, USA [10], and the Children’s Medical Centre in Texas, USA [8], had 235 respondents (78% response rate) and 33 respondents (83% response rate) respectively. In comparison, this study had a smaller targeted sample, but 100% participation. There were limited unanswered questions by individuals, with the exception of a question on confidence in examining for dysphagia; possible reasons could be that nurses did not examine this condition or were unsure what it meant. Moreover, both previous surveys did not ask pediatric oncology nurses specifically about perceived barriers; our study is likely the first to do so.

Most participants were experienced and many worked in pediatric oncology over 6 years. However, the majority had no specific training or CPE in oral healthcare. Such inadequacy in oral health-related training has also been observed in other pediatric oncology nurses [10], critical care nurses [9, 13], and adult general and oncology nurses [7]. For instance, the Pennsylvania study had about 75% of respondents with 3 hours of oral health-related education; about 60% did not have competency regarding assessment of teeth and gums in nursing schools [10]. The upward trends in overall knowledge scores after attending ≤3 hours of CPE was not statistically significant. CPE could be useful for increasing knowledge scores, but we need a bigger sample size to prove this, and ascertain if number of hours matters. None in this group had >3 hours of CPE.

The lack of training could have contributed to the knowledge gap, as no one answered all knowledge questions correctly. Currently, the only national nursing guidelines on nursing management of oral hygiene readily available may be outdated and is not specific to children or oncology care [14]. Hence, a pediatric oncology evidence-based oral nursing guideline is needed. The majority did not know of any international guidelines on oral care for pediatric oncology patients, despite the information being readily available online. Past research has found that only a minority of critical care nurses would refer to hospital policies/guidelines [13]. Instead, most relied on previous experience or basic nursing training as a primary source of information on oral healthcare [9, 15]. However, in our study, experience (years as pediatric oncology nurse) did not improve oral health knowledge scores. This underlines the importance of including formalized oral health training as part of nursing training programs. The importance of CPE beyond nursing school and in inter-disciplinary cooperation with dental professionals have also been highlighted by past surveys [6–8].

Based on the knowledge answers, participants had high general awareness of the importance of oral health. However, there were knowledge gaps in the more specific oral hygiene and dietary habits conducive for oral health. Less than three-quarters knew that fluoride toothpaste should be used, and less than half knew that toothbrushing should continue regardless of platelet counts [11]. In addition, most were unaware that the frequency of sugary intake is more crucial than the amount of sugar intake in causing decay. These knowledge results corroborated with the practice frequencies for recommendations of fluoride toothpaste use (74.2%) and advice to reduce sugar intake (51.7%). Foam brush, toothbrush and lip balm were the most common oral aids our participants would recommend, similar to the Texas study [8]. However, there was no consensus regarding the use of other oral healthcare aids. A local oral care protocol may help bridge knowledge gaps and ensure correct oral hygiene and dietary recommendations are made to patients/parents.

It is heartening that a high proportion (>90%) of nurses felt confident to assist with oral care including toothbrushing, and did not find it an unpleasant task, unlike findings in other countries’ studies [6–8]. This difference may be attributed to our study examining paediatric oncology nurses, whereas Ohrn [6] and Southern [7] surveyed general oncology nurses. In addition, Tewogbade [8] and Southern [7] did not survey nurses on their direct provision of oral care, and this may be due to oral care falling outside their scope of responsibility, given that care assistants in the US typically adopt the responsibility of such tasks. Conversely, nurses in Singapore may feel that oral care is part of their job scope and thus not find it an unpleasant task. This theory was also evident in a previous survey of Singapore critical care nurses [9], with only 0.4% citing oral care provision to be an “unpleasant task” (Table 3).

However, in practice, fewer nurses in the present study (85%) would advise patients to brush and only 65% would assist patients to do so. This discrepancy is likely attributed to poor patient cooperation, the main barrier highlighted by 90.5% of nurses. This was also the main barrier corroborated in another local study on critical care nurses (Table 3) [9]. This highlights that apart from structured training and oral care protocols to standardize and increase the frequency of evidence-based oral care, local nursing training also needs to address strategies to manage the uncooperative patient, to target improvement of the practical delivery itself. Effort should be invested in tools or means to improve competence in oral care provision e.g. behavioral strategies for uncooperative patients and effective brushing techniques. These would further empower nurses who are willing to take on the responsibility but struggle in delivering oral care.

Oral care examinations performed were inconsistent and incomplete. While most were confident in examining the mouth for simple or common conditions e.g. oral pain, mucositis, ulcers, and cold sores, they were less confident in examining more severe complications e.g. trismus and dysphagia. Such phenomenon is similar in past studies [8, 10], highlighting the need to train nurses to identify common oral diseases and complications in children with cancer. This is further reinforced by past research that demonstrated the correspondence between the level of confidence in oral health knowledge and frequency of oral examination [16].

It is likely that the environment and equipment in hospital wards are not the most conducive for comprehensive oral examinations, with the Texas study reporting about half of pediatric oncology nurses using just room light for oral examination [8]. Hence, in addition to improving nurses’ knowledge of oral conditions, encouraging referral for dentists’ diagnosis and management should be done in tandem.

Most knew the right timing to refer but few had initiated referrals, with more than half citing that they have no authority or responsibility to do so. This might be related to local policies and practices where doctors are deemed responsible for referrals. Nonetheless, as team members who have an active role in oral examination and care for patients in the wards, nurses can remind doctors. This is in line with Perry et al.’s conclusions that a form of ‘inter-professional collaboration’ is important between nurses and doctors to improve patients’ oral health [10]. Another major barrier to referral was parents’ notions of high cost and long waiting time for dental treatment. Most nurses were unaware of an existing in-hospital dental referral letter for oncology patients; a clearer referral workflow and responsibility outline is needed. Workflows could also be modified to facilitate necessary dental appointments.

This study has several limitations. Firstly, it was conducted in one study center with small sample size, limiting generalizability. However, all the center’s eligible oncology nurses responded, possibly attributed to response enhancement measures (verbal reminders), thereby reducing non-response bias [17]. Secondly, the data collected was based on the nurses’ self-reported attitudes and practices which could be subjected to recall bias [18], and social desirability bias [19]. Participants may not have recalled their actual training and practices accurately, or be compelled to report attitudes and practices more favorably. Thirdly, the questionnaire used was developed by the study team and has not been formally validated. Nonetheless, this study provided insights about the oral healthcare competencies and attitudes of pediatric oncology nurses, as well as gaps and barriers in oral healthcare provision.

Finally, this survey was administered before the COVID-19 pandemic. The future implementation of training and provision of equipment should account for possible concerns of aerosol generated from oral care e.g., appropriate personal protection equipment should be provided. A follow-up study may help identify if COVID-19 presents new barriers and can also involve multiple centers to increase sample size. Qualitative studies may also help to explore what further assistance nurses require. Future research could also examine barriers to oral care faced by patients/caregivers, or oncology doctors’ referral practices, and parental/patient preferences to dental attendance.

## Conclusions

Most nurses believed that they have a role in helping to maintain patients’ oral health, despite deficiencies in oral health-related knowledge, perceived abilities, attitudes, and practices. Different barriers exist in patient education, oral care provision, oral examination, and dental referral. Hence, these findings are useful to guide development of an evidence-based inter-disciplinary training program and oral care guidelines for pediatric oncology nurses. Didactic training and guidelines should target the worst gaps in knowledge e.g. oral hygiene and dietary advice. CPE is recommended for periodic knowledge reinforcement/update. Practical training should focus on empowering nurses to identify common oral diseases and complications, with complementary guidelines on oral examination and referral criteria. A clearer referral workflow and responsibility outline may also improve referral practice, where beliefs that it is the doctors’ responsibility to refer, perceived parental reluctance, and poor awareness of existing resources are key challenges. Existing resources e.g. hospital dental service and referral templates should be made known. Finally, practical support is recommended (e.g. provision of oral aids in wards and specific allocation of time for oral nursing) to address barriers in assisting toothbrushing, that is largely attributed to the lack of time or physical aids and patient cooperation.

## Supplementary information

Supplementary Information

## Data Availability

Data is available on request from the authors.
