# Supplementary material for: A survey of pediatric oncology nurses’ oral health knowledge, attitudes, practices, and perceived barriers in a Singapore Tertiary Children’s Hospital
Source: BDJ Open. 2023 Feb 7;9:3. doi: 10.1038/s41405-023-00130-2 (PMC9902813; doi:10.1038/s41405-023-00130-2)
Supplement: Supplementary file 1 — Supplementary Information [file 41405_2023_130_MOESM1_ESM.docx]

**Supplemental Table S1.**

*Demographics and Professional Characteristics of Participants (n=63)*

|  | n | % |
| --- | --- | --- |
| **Gender**  Female | 63 | 100 |
| **Job position**  Enrolled Nurse  Registered Nurse – Staff Nurse  Registered Nurse – Senior Staff Nurse/ Assistant Nurse Clinicians  Registered Nurse – Nurse Manager/Clinicians  Registered Nurse – Advanced Practice Nurse | 9  14  37  3  0 | 14.3  22.2  58.7  4.8  0.0 |
| **Years working as nurse**  ≤5years  6 to 10 years  ≥11 years | 19  25  19 | 30.2  39.7  30.2 |
| **Years working in a paediatric oncology ward**  ≤5years  6 to 10 years  ≥11 years | 29  25  9 | 46.0  39.7  14.3 |
| **Highest level of nursing education**  National Institute of Technical Education Certificate  Diploma  Bachelor  Master | 1  12  48  2 | 1.6  19.0  76.2  3.2 |
| **Has undergone specialty training – Advance diploma or equivalent**  No  Yes  Paediatric  Oncology  Others | 38  3  18  4 | 60.3  4.8  28.6  6.3 |
| **Number of hours of training related to oral health care received basic nursing training**  0 hour  0.5-10 hours | 37  26 | 58.7  41.3 |
| **Has clinical competency regarding the assessment of the teeth and gums during nursing training**  No  Yes  Question left blank | 46  14  3 | 76.7  23.3  - |
| **Has taken Continuing Professional Education Courses relating to oral health care for paediatric oncology patients in the past 5 years**  No  <3 hours  > 3 hours  Question left blank | 41  19  0  1 | 68.3  31.7  0.0  - |

**Supplemental Table S2.**

*Oral Conditions That Nurses Evaluated in Children with Cancer.*

| **Oral conditions** | **n (%)** |
| --- | --- |
| Oral mucositis | 98.4%, n=62 |
| Oral ulcerations | 87.3%, n=55 |
| Oral bleeding | 85.7%, n=54 |
| Swollen gums | 79.4%, n=50 |
| Oral pain | 79.4%, n=50 |
| Dental cavities | 49.2%, n=31 |
| Inflamed soft tissues | 46.0%, n=29 |
| Trouble with swallowing | 44.4%, n=28 |
| Fungal infection | 39.7%, n=25 |
| Clinical abscess | 36.5%, n=23 |
| Dental plaque | 34.9%, n=22 |
| Bad breath | 30.2%, n= 19 |
| Oral pathology | 6.3%, n=4 |
|  | |

**Percentages do not add up to 100% because multiple choices were allowed.*

**Supplemental Figure S1.**

*Oral Health Care Aid Advised by Nurses.*

**Supplemental File S1.**

*Nurses survey questionnaire*

**Screening Questions**

1. Are you currently employed in Singapore as a Paediatric Oncology or Paediatric Haematology/Oncology Nurse?

α Yes  No

1. Do you provide direct patient care?
   α Yes  No

If you answered *NO* to either of the two questions above, please do not continue this survey. Thank you very much for your time.

If you answered *YES* to both of the questions above, please continue the survey below.

**(A) Demographics**

1. I am a:

- Enrolled Nurse
- Registered Nurse – Staff Nurse
- Registered Nurse – Senior Staff Nurse/ Assistant Nurse Clinicians
- Registered Nurse – Nurse Manager/Clinicians
- Registered Nurse – Advanced Practice Nurse

1. How many years have you been working as a nurse?

- > 1 year
- 1 to 5 years
- 6 to 10 years
- 11 to 15 years
- ≥ 16years

1. How many years have you been working in a Paediatric Oncology ward?

- > 1 year
- 1 to 5 years
- 6 to 10 years
- 11 to 15 years
- ≥ 16years

1. What is your highest level of nursing education?

- NITEC
- Diploma
- Bachelor
- Master

1. Undergraduate training country?

- Singapore
- Others (please specify):____________________

1. Have you undergone specialty training – Advance diploma or equivalent?

- Yes,
  - Paediatric
  - Oncology
  - Others (please specify):____________________
- No

1. Approximately, how many hours of education and/or training related to oral health care did you receive in Nursing School?

A) Basic Training: _____hours

B) Advanced Training: _____hours

1. During your nursing education and/or training, did you have any clinical competency regarding the assessment of the teeth and gums?

- Yes
- No

1. In the past 5 years, have you taken any Continuing Professional Education (CPE) Courses relating to oral health care for paediatric oncology patients?

- No
- 3 hours or less
- Between 3 and 5 hours
- More than 5 hours

**(B) Knowledge**

1. **Oral Health and Oncology**
2. The oral cavity is the most common documented source of sepsis in the immunocompromised oncology patient.

 True Δ False ε Don’t know

1. Children undergoing radiation or chemotherapy could potentially develop oral complications, such as: oral mucositis/stomatitis, oral candidiasis infections, herpes simplex virus infections (cold sores), dental caries (cavities), as a result of oncology treatment.
    True Δ False ε Don’t know
2. Good oral hygiene is important to prevent and/or reduce the severity of oral mucositis in paediatric oncology patients.

 True Δ False ε Don’t know

1. How often should the mouth of paediatric oncology patients, undergoing active cancer therapy, be assessed by caregivers (i.e. parents, guardians, nurses, and/or physicians)?
    Daily Δ Weekly ε Monthly ε Other (please specify) ________ ε Don’t know
2. Are there any guidelines (local or international) on oral health care protocol in paediatric oncology patients that you are using/aware of?

 Yes , if so, name of guideline: ___________  No

1. It is recommended that paediatric oncology patients be referred to a dentist for consultation _________ cancer treatment, in order to optimize oral health of paediatric oncology patients undergoing cancer therapy.
    Before
    During
    After
    All of the above
    None of the above
    Don’t know
2. **Oral Habits – Fluoride and Toothbrushing**
3. Toothbrushing should continue regardless of the child’s:

A) Red blood cell counts  True Δ False ε Don’t know

B) White blood cell counts  True Δ False ε Don’t know

C) Platelet counts  True Δ False ε Don’t know

1. Oncology patients should brush their teeth at least twice a day.

 True Δ False ε Don’t know

1. Paediatric oncology patients should use a ________ bristled toothbrush.
    Soft Δ Medium ε Hard ε Other (please specify) _________ ε Don’t know
2. Oral swabs can be used for oral hygiene as a long-term substitute for toothbrushing during oncology therapy.

 Strongly Disagree  Disagree  Neutral Δ Agree ε Strongly agree

1. Paediatric oncology patients should use ___________ toothpaste.
    Fluoridated
    Non-fluoridated
    None, as Paediatric oncology patients should not use toothpaste
    Other (please specify) ____________________
    Don’t know
2. **Diet**
3. The total amount of sugar ingested/consumed is a greater risk factor for tooth decay than the frequency of sugary food/beverages.
    Strongly Disagree  Disagree  Neutral Δ Agree ε Strongly agree

**(C) Attitudes/Beliefs**

1. Oncology nurses play an important role in maintaining patients’ oral health

 Strongly Agree
 Agree Somewhat
 Neutral
 Disagree Somewhat
 Strongly Disagree

1. Do you think good oral hygiene is important to oncology patients?
    Yes
    No
2. How important is it for you to ensure that your patients brush their teeth at least twice a day?
    Very important  Somewhat important  Neutral
    Not really  Not at all
3. If the patients/parents do not brush the patient’s teeth, how important is it for you to help brush the child’s teeth?

 Very important  Somewhat important  Neutral
 Not really  Not at all

1. Do you think that nurses need further training to provide oral hygiene education to patients and caregivers?
    Yes  No

**(D) Perceived Practices/Abilities**

1. **Practices**

**Referring Practices**

1. Who would usually refer newly diagnosed paediatric oncology patients to in-house dentists?

 Nurses

 Doctors

 Others: (Specify)____________

1. Is there a referral form template that you use to refer patient to the in-house dentists of KKH?
    Yes  No

**Oral Assessment Practices**

1. How frequently/when do you examine the oral cavity of your paediatric oncology patient? Please tick all that apply.
    Every shift (every 8 hours)
    More frequently than every 8 hours
    Daily
    Upon admission
    Prior to start of chemotherapy/radiotherapy
    After chemotherapy/radiotherapy
    At presentation of symptoms
    At request of patient/parent
    Immediately prior to discharge
    No examination performed
2. Does your department have a standardised oral assessment scale/form that you use?

 Yes , if so, name of form/scale: ___________  No

1. Which of the following oral conditions do you evaluate during your oral examination? Please tick all that apply.
    Mucositis
    Bleeding
    Swollen gums
    Ulcerations
    Oral pain
    Inflamed soft tissue
    Clinical abscess
    Trouble swallowing
    Fungal infection
    Plaque
    Bad breath
    Cavities
    Oral pathology
    Other conditions (please specify): ________________

**Advice on Diet, Toothbrushing, Fluoride Toothpaste, Mouthrinse**

1. How often do you advise your patient to eat/drink sugary food/drinks less frequently?

 Always (for all my patients)
 Often (for more than half of my patients)
 Seldom (only for a few of my patients)
 Never

1. How often do you remind your patients to brush their teeth?
    Always (for all my patients)
    Often (for more than half of my patients)
    Seldom (only for a few of my patients)
    Never
2. How often do you assist your patients to brush their teeth?
    Always (for all my patients)
    Often (for more than half of my patients)
    Seldom (only for a few of my patients)
    Never
3. What toothpaste do you advise your patients to use?

 Fluoridated
 Non-fluoridated
 Other (please specify) ____________________
 None, as Paediatric oncology patients should not use toothpaste

1. Which of the following oral care aids do you instruct parents to use? (Please tick all that applies)
    Oral swab stick/foam brush
    Soft bristled toothbrush

 Medium bristled toothbrush

 Hard bristled toothbrush
 Toothpaste
 Mouthwash
 Wash cloth
 Dental floss
 Water
 Lip balm
 None
 Others (please specify): _________________

1. **Perceived Abilities/Confidence in Oral Assessment and Care**
2. How confident are you about advising parents on their child’s:
   1. Oral hygiene home care (e.g. tooth brushing)?
       Very confident
       Somewhat confident
       Neutral
       Not really confident
       Not confident at all
   2. Dietary habits to prevent tooth decay?
       Very confident
       Somewhat confident
       Neutral
       Not really confident
       Not confident at all
   3. Usage of fluoridated toothpaste?
       Very confident
       Somewhat confident
       Neutral
       Not really confident
       Not confident at all
3. How confident are you in your ability to do the following? Select one response per row.
   1. Examine health of teeth and/or gums
       Very confident  Not at all confident  Somewhat confident
   2. Identify signs of tooth decay in a child

 Very confident  Not at all confident  Somewhat confident

- 1. Examine for the presence of oral appliances (i.e. braces, retainer, dentures)
      Very confident  Not at all confident  Somewhat confident
  2. Examine for the presence of oral pathology (i.e. oral mucositis/stomatitis, oral candidiasis, cold sores, ulcers, etc.)
      Very confident  Not at all confident  Somewhat confident
  3. Examine if the patient experiences trismus (difficulty opening mouth)
      Very confident  Not at all confident  Somewhat confident
  4. Examine if the patient experiences dysphagia (difficulty swallowing)

 Very confident  Not at all confident  Somewhat confident

- 1. Examine if the patient experiences xerostomia (dry mouth)
      Very confident  Not at all confident  Somewhat confident
  2. Examine for the presence of oral pain
      Very confident  Not at all confident  Somewhat confident
  3. Provide oral hygiene care instruction
      Very confident  Not at all confident  Somewhat confident
  4. Discuss the importance of seeking professional dental care regularly
      Very confident  Not at all confident  Somewhat confident
  5. Provide instruction pertaining to managing oral complications/conditions
      Very confident  Not at all confident  Somewhat confident
  6. Other: ________________________________________________________
      Very confident  Not at all confident  Somewhat confident

1. Please indicate the extent to which you agree or disagree with each of the following statements. Select one response per row.
   1. I am comfortable performing oral care procedures on patients.
       Strongly Agree
       Agree Somewhat
       Neither Agree or Disagree
       Disagree Somewhat
       Strongly Disagree
   2. I am adequately trained to provide oral health care instructions/education to patients.
       Strongly Agree
       Agree Somewhat
       Neither Agree or Disagree
       Disagree Somewhat
       Strongly Disagree
   3. I am adequately trained to provide oral care procedures, i.e. assisting with tooth brushing, mouth swabbing, and/or application of oral topical medications, on patients.
       Strongly Agree
       Agree Somewhat
       Neither Agree or Disagree
       Disagree Somewhat
       Strongly Disagree
   4. I am adequately trained to perform oral examinations on patients.
       Strongly Agree
       Agree Somewhat
       Neither Agree or Disagree
       Disagree Somewhat
       Strongly Disagree

**(E) Barriers**

1. Some of the concerns preventing you/your team from referring your patients to see a dentist are:
   1. Do not have authority to do so as it is not within my job scope to refer
       Strongly disagree
       Disagree
       Neither agree nor disagree
       Agree
       Strongly agree
   2. Do not know enough about dental conditions to refer correctly
       Strongly disagree
       Disagree
       Neither agree nor disagree
       Agree
       Strongly agree
   3. It is the responsibility of the oncology doctor to refer
       Strongly disagree
       Disagree
       Neither agree nor disagree
       Agree
       Strongly agree
   4. Do not know who to refer to
       Strongly disagree
       Disagree
       Neither agree nor disagree
       Agree
       Strongly agree
   5. No time to do dental evaluation
       Strongly disagree
       Disagree
       Neither agree nor disagree
       Agree
       Strongly agree
   6. Parents perception that baby teeth are not important
       Strongly disagree
       Disagree
       Neither agree nor disagree
       Agree
       Strongly agree
   7. Parents perceived long waiting time for dental treatment
       Strongly disagree
       Disagree
       Neither agree nor disagree
       Agree
       Strongly agree
   8. Parents’ perceived high cost of dental care
       Strongly disagree
       Disagree
       Neither agree nor disagree
       Agree
       Strongly agree
   9. Others (please specify): ________________________________
       Strongly disagree
       Disagree
       Neither agree nor disagree
       Agree
       Strongly agree
2. Barriers that hinder me from performing oral care for the patients are (please tick all that apply):
    The patient is uncooperative

 The patient is very unwell
 The patient has a sore mouth

 The patient is intubated

 Inadequate staffing
 Lack of oral healthcare aids e.g. toothbrushes, toothpastes
 Lack of knowledge

 Not sure what to look out for
 It is an unpleasant task

 I have other more important tasks

 Lack of time

 The parents are the ones responsible for the patient’s oral care

 The doctors are the ones responsible for the patient’s oral care

 Others (please specify): ______________________________

____________________________________________________________________________________________________________________________________________________________________________________________________________________________________________________________________________
